# Validation of the Brief Perceived Positive Lockdown Impact Scale PPLIS-4

**DOI:** 10.3390/ijerph192013198

**Published:** 2022-10-13

**Authors:** Aleksandra M. Rogowska, Dominika Ochnik, Karolina Chilicka, Iuliia Pavlova, Cezary Kuśnierz

**Affiliations:** 1 Institute of Psychology, University of Opole, 45-052 Opole, Poland; 2 Faculty of Medicine, University of Technology, 40-555 Katowice, Poland; 3 Department of Health Sciences, University of Opole, 45-040 Opole, Poland; 4 Department of Theory and Methods of Physical Culture, Lviv State University of Physical Culture, 79007 Lviv, Ukraine; 5 Faculty of Physical Education and Physiotherapy, Opole University of Technology, 45-758 Opole, Poland

**Keywords:** cross-cultural validation study, emerging adults, factor analysis, the positive impact of the COVID-19 pandemic, university students, well-being

## Abstract

Background: Although research showed that positive aspects of the lockdown were perceived during the pandemic, there are no tools to test the positive impact of mandatory social isolation on life. The present study aims to validate a newly developed, brief, four-item perceived positive lockdown impact scale (PPLIS-4). Methods: A cross-sectional online survey study was formed among 4370 adults in three samples: Sample 1 consisted of university students from Poland and Ukraine, Sample 2 consisted of Polish university students under 26 (emerging adults), and Sample 3 consisted of Polish and Ukrainian adults above 25 (non-emerging adults). The standardized questionnaire was used for criterion validity to measure life satisfaction (SWLS), perceived stress (PSS-10), anxiety (GAD-7), and depression (PHQ-9). Results: The exploratory factor analysis (EFA) showed a one-factor solution in Sample 1 in Polish and Ukrainian university students. The confirmatory factor analysis (CFA) and confirmatory composite analysis (CCA) showed the one-factor structure appropriate for the PPLIS-4 among emerging and non-emerging adults. Criterion validity was also confirmed since the PPLIS-4 was positively related to the SWLS and negatively related to stress, anxiety, and depression. Conclusions: The PPLIS-4 is a short but valid questionnaire to assess the positive aspects of lockdown. The PPLIS-4 can be used during the COVID-19 pandemic to measure some positive effects of changes in lifestyle as an aspect of resilience.

## 1. Introduction

The COVID-19 pandemic impacted the mental health and well-being of populations worldwide [1,2]. Studies showed that stress, anxiety, and depression increased significantly during the subsequent pandemic waves [3,4,5,6]. Gender differences were shown during the pandemic, with women experiencing higher levels of stress, anxiety, and depression than men [7,8,9,10,11]. Additionally, young adults [12,13,14,15,16], particularly university students, were found to be the most vulnerable population [7,8,9,17,18,19]. Indeed, emerging adulthood is a challenging period related to self-focus, the feeling of being between adolescence and adulthood, optimistic thinking about the future, the exploration of identity, and instability within social roles [20]. Research showed that mental health and life satisfaction significantly declined among emerging adults during the COVID-19 pandemic due to limited peer contact, worsening financial situations, and returning to the parental home from dormitories [21]. However, other research showed no changes in mental health among emerging adults [22], or even better mental health and well-being during the first lockdown compared to the pre-pandemic period [23]. Therefore, more research is required to explain these inconsistencies among emerging adults during the pandemic. 

The adverse consequences of the COVID-19 pandemic were not the only ones demonstrated. Resilience was also noted since restriction-related changes in lifestyle, and various difficulties became challenges for some people to survive and improve their quality of life [24,25]. Adaptation to changing life patterns forced new habits to promote healthy behavior, such as maintaining personal hygiene, increasing physical activity, eating a healthy diet, or consuming less alcohol or substances [26,27,28,29,30,31]. Spending more time with family and friends during the lockdown, improving parenthood and relationship quality, and increasing donations and solidarity positively impacted human life during the pandemic [26,28,29,30,32,33]. Business growth and innovations were observed in many sectors, particularly in handicraft and domestic production, as well as areas related to online business, informatics, new digital technologies, and online software, like Zoom, video games, services for distance learning and working, and telemedicine [26,32]. Many people reported increased activity in realizing interests and hobbies (creativity, music, art, reading books, watching films), learning new skills, and exploring new technology during social isolation [27,29,32]. However, Sudo [34] showed that disparities in economic status contributed to lifestyle changes, increasing the positive effects of the pandemic among socially advantaged people, but decreasing well-being in socially disadvantaged individuals.

Although the previous research showed that some people found some positive aspects of the COVID-19 pandemic, little is known about whether those individuals showed better mental health and well-being than persons who did not perceive any positive impact of the pandemic on their life. The lack of such research is related to the lack of measurement methods for assessing the perceived positive impact of the pandemic on human life. Previously mentioned studies [26,27,28,29,30,31,32,33,34] used self-developed single questions, which were never validated. The present study demonstrates the validation of a newly developed short measure to assess the perceived positive lockdown impact on life during the COVID-19 pandemic. Since young people are the group most vulnerable to mental health issues, the study will be validated with the newly developed scale in the sample of emerging adults in comparison to older adults. Furthermore, country and gender differences will be examined to verify the structure of the scale in a cross-cultural context. The criterion validity will be tested on dimensions of well-being. We expect higher scores on the perceived positive impact of lockdown will be related to higher life satisfaction and lower stress, anxiety, and depression levels. 

## 2. Materials and Methods

### 2.1. Study Design and Procedure

Three cross-sectional online survey studies were performed in Poland and Ukraine during the COVID-19 pandemic. Three samples were recruited at different times. 

#### 2.1.1. Sample 1

The study in Sample 1 was performed in November 2020 during the second pandemic wave and included Polish and Ukrainian university students. The inclusion criteria consisted of being a university student and being above 18 years old. Individuals from Poland were recruited from the Opole University of Technology and the University of Opole between 3 and 27 November 2020. Ukrainian participants were recruited from the Lviv State University of Physical Culture, Lviv Polytechnic National University, and Ternopil Volodymyr Hnatiuk National Pedagogical University between 2 and 26 November. In both countries, classes were performed at universities remotely online, using e-learning services (like Moodle or Teams). The restriction levels during the data collected were assessed using the Stringency Index (SI, ranging from 0 to 100), indicating SI = 75.19 for Poland and SI = 60.35 for Ukraine [35]. Online learning was performed in November 2020, so university teachers encouraged students to participate in the study during their online classes. Students were invited to share the link to the survey using the Moodle and Teams online educational platforms. Participants were also invited to the study via university students’ groups on Facebook, Viber, and Telegram. The studies were anonymous and voluntary. Participants’ personal data was not obtained, and no compensation was offered as an incentive to participate. The minimal sample size for factor analysis is 100 people. However, 300 is considered a good size, 500 is very good, and 1000 or more is excellent [36]. Among Ukrainian students, 1713 responded to the invitation, but 64 refused, so the final sample consisted of 1649 individuals. Among Polish university students, 1699 responded to the invitation, but 118 did not agree, so the final sample included 1581 individuals. The total of Sample 1 consisted of 3230 university students from both countries.

#### 2.1.2. Sample 2

Study Sample 2 was recruited between 14 April and 16 June 2021 among university students from the Opole University of Technology (Poland) during the third wave of the COVID-19 pandemic. At that time, classes were performed online at the university, and the restriction level was SI = 63.57 [35]. The inclusion criteria consisted of being a university student and being between the ages of 18 and 25 (as a criterion of an emerging adult). Similar to the previous study, e-learning was conducted during the third wave of the COVID-19 pandemic; therefore, the invitation to the survey was disseminated during online classes and via social media in the same way as in the previous study. Initially, 482 people responded to the invitation, but nine refused, and 25 did not meet the age criteria, so the final sample included 448 emerging adults.

#### 2.1.3. Sample 3 

Study Sample 3 was recruited during the second wave of the COVID-19 pandemic among adults from Poland (from 19 November 2020 to 15 January 2021) and Ukraine (from 14 December 2020 to 17 February 2021). The restriction level (SI) was 75.19 and 57.54 in Poland and Ukraine, respectively [35]. The exclusion criterion was being under the age of 26 (representing emerging adults). The online survey was disseminated using the snowball technique among friends, family members, and private groups on Facebook. Additionally, the authors of the study shared links to an online survey form via personal and institutional emailing lists, primarily among colleagues, namely administrative and academic staff at the Opole University of Technology, the University of Opole, the University of Technology in Katowice from Poland, and the Lviv State University of Physical Culture from Ukraine. Among the 743 people who answered the invitation, seven refused the study, while 84 were excluded because they did not meet the criteria (i.e., they were aged below 26). Therefore, 652 non-emerging adults were included in the final sample.

### 2.2. Measurement

The online survey was prepared using Google Forms and consisted of several parts, like (1) information about the study and informed consent; (2) demographic information; (3) a newly developed scale of perceived positive lockdown consequences; (4) standardized questionnaires to measure life satisfaction, perceived stress, anxiety, and depression. The survey was developed in Polish and English, and then translated into Ukrainian and back-translated due to the guideline for translation and cross-cultural adaptation [37,38]. No missing data were reported in Sample 1, Sample 2, and Sample 3, as all responses were mandatory in the Google form.

#### 2.2.1. Perceived Positive Lockdown Impact

Ochnik et al. [39] developed the Perceived Positive Lockdown Impact Scale (PPLIS) to assess the positive aspects of compulsory social isolation during the COVID-19 pandemic-related lockdown. The question on the scale was, “I think that the situation associated with the coronavirus pandemic (COVID-19) has positively affected my life through…” Participants rated on a 7-item Likert scale (from 1 = *Strongly disagree* to 7 = *Strongly agree*) the extent to which the current situation associated with the Coronavirus pandemic may positively affect their lives in the following four aspects: (1) spending more quality time with family; (2) spending more quality time with friends; (3) spending more time relaxing and on entertainment; (4) spending more time on personal development and interests (hobby). The PPLIS is presented in Appendix A, Table A1 (in English), Table A2 (in Polish), and Table A3 (in Ukrainian). The higher the PPLIS scores, the more positive consequences were perceived during the pandemic. The psychometric properties and validation of the PPLIS are presented in the following parts of the article.

#### 2.2.2. Satisfaction with Life

The Satisfaction with Life Scale (SWLS) was developed by Diener et al. [40] to measure global cognitive judgments concerning how much a person is satisfied with their life. The scale includes five items and a seven-point Likert scale (from 1 = *Strongly disagree* to 7 = *Strongly agree*). Total scores range from 5 to 35. Higher scores indicate a higher level of life satisfaction. The internal consistency (Cronbach’s α) of the SWLS was high and ranged from 0.79 to 0.89 in various studies [41]. In the current study, Cronbach’s α was 0.83 in Sample 1. 

#### 2.2.3. Perceived Stress

The Perceived Stress Scale (PSS-10) was developed for a global assessment of psychological stress [42]. The questionnaire consisted of ten items. Respondents indicated how often they experienced a given behavior in the month preceding the test, using a 5-point Likert scale (ranging from 0 = *Never* to 4 = *Very often*). Higher scores mean a higher level of perceived stress. The internal consistency of the PSS-10, assessed by Cronbach’s α, was 0.78 in the previous study [43], and currently, in Sample 1, Cronbach’s α = 0.86.

#### 2.2.4. Anxiety

The 7-item Generalized Anxiety Disorder (GAD-7) scale was developed to assess anxiety risk, considered as a state, in the general population, consistent with the Diagnostic and Statistical Manual of Mental Disorders, fifth edition (DSM-V) criteria [44]. Participants self-rated how often they experienced anxiety symptoms in the two weeks preceding the test using a 4-point Likert scale (0 = *Not at all*, 1 = *Several days*, 2 = *More than half the days*, and 3 = *Nearly every day*). Scores above 10 points indicate an anxiety disorder risk. The Cronbach’s α for the GAD-7 in the original study was 0.92 [44], and 0.92 in Sample 1.

#### 2.2.5. Depression

Depression symptoms were assessed using the Patient Health Questionnaire (PHQ-9) developed by Kroenke et al. [45] to screen for depression symptoms according to DSM-V diagnostic criteria. Participants used a Likert-type response scale (ranging from 0 = *Not at all* to 3 = *Nearly every day*). A cutoff score of 11 or above is recommended for screening a major depressive disorder risk. The internal consistency reliability of the original version measured by Cronbach’s α equals 0.86 [46]. The Cronbach’s α = 0.89 was found in this study for Sample 1.

### 2.3. Participants

Sample 1 consisted of 3230 university students, aged between 18 and 59 (*M* = 21.40, *SD* = 3.46), including 1581 individuals from Poland (48.95%) and 1649 from Ukraine (51.05%). In the sample, 1912 (59.20%) were women, and 1318 (40.80%) were men. Participants represented various faculties of technical and humanistic university types, including economy and political sciences (e.g., social communication, human resources and management, economy, politics, and law), engineering (automatics, mechanical engineering, electrical engineering, logistic, architecture and construction, energy, and transport technologies), health sciences (nursing, cosmetology, emergency, physiotherapy, physical education, and tourism and recreation), humanities (e.g., languages, fine arts, archeology, culture, and history), information technologies (e.g., computer sciences and informatics), social sciences (e.g., sociology, psychology, and pedagogy), and science (e.g., chemistry, biology, geography, math, and physics). Individuals studied at bachelor (82.90%), master (16.94%), and postgraduate or doctoral (0.16%) levels, representing the first (34.24%), second (31.05%), third (19.98%), fourth (12.20%), and fifth (3.53%) year of study. Participants studied full-time (91.77%) and part-time (8.23%). 

Sample 2 included 448 emerging adults, aged between 19 and 25 (*M* = 21.53, *SD* = 1.51), including 237 women (52.90%) and 211 men (47.10%). Participants represented all faculties of the Opole University of Technology, including civil engineering and architecture (12.72%), economics and management (22.54%), electrical engineering, automatic control and informatics (19.20%), production engineering and logistics (3.79%), mechanical (4.91%), and physical education and physiotherapy (36.83%). Participants studied at bachelor’s (69.20%), master’s (14.73%), and postgraduate or doctoral (16.07%) levels, and they represented first the (34.38%), second (34.38%), third (23.88%), fourth (5.58%), and fifth (1.79%) year of study. The sample included full-time (92.86%) students and part-time (7.14%) studies. 

Sample 3 consisted of 652 adults ranging in age between 26 and 72 (*M* = 41.56, *SD* = 9.50) from Poland (*n* = 332, 50.90%) and Ukraine (*n* = 320, 49.10%). In the sample, 450 (69.00%) were women, 123 (18.90%) were single, and 490 (75.20%) had children. Mean work experience (seniority) was 17 years (ranging from 0 to 53 years, *M* = 17.28, *SD* = 9.68). Participants worked in various job sectors, including higher education (*n* = 372, 57.06%), administration (*n* = 101, 15.49%), primary and high school education (*n* = 41, 6.29%), services (*n* = 27, 4.14%), healthcare (*n* = 24, 3.68%), uniformed service (*n* = 19, 2.91%), production (*n* = 13, 1.99%), and management (*n* = 12, 2.15%), trade (*n* = 9, 1.38%), library (*n* = 8, 1.23%), logistics (*n* = 8, 1.23%), and engineering and mechanics (*n* = 3, 0.46%). Nine participants (1.38%) were unemployed, one was on maternity leave, and one retired. 

### 2.4. Statistical Analysis

Descriptive statistics were calculated, including a range of scores, mean (*M*), standard deviation (*SD*), median (*Mdn*.), skewness, and kurtosis. Pearson’s correlations were calculated to examine associations between variables. 

The structural validation was tested using exploratory factor analysis (EFA), with the principal components estimation method (without rotation). EFA was performed in Sample 1 twice: in Polish (*n* = 1581) and Ukrainian (*n* = 1649) groups of university students. We replicated the EFA to establish the factor structure of the PPLIS across countries. The correlation matrix between the items of the PPLIS, the Kaiser–Meyer–Olkin (KMO), and Bartlett’s test of sphericity were initially performed to explore the data’s properties. Subsequently, the CFA with maximum likelihood (ML) estimation method was conducted in Sample 2 of Polish university students under 26 as an emerging adults group (*n* = 448) and replicated in Sample 3 of adults above 25 years old as a non-emerging adults group (*n* = 652). All structural models were evaluated using several goodness-of-fit criteria, including ML χ^2^, df and *p*-value, standardized root mean squared residual (SRMR ≤ 0.08 is acceptable), root mean square error of approximation (RMSEA; acceptable fit if ≤0.08, adequate fit if <0.06, and good if <0.04), and comparative fit index (CFI is acceptable if ≥0.90, and good if >0.95) [47]. The measurement invariance (MI) was examined in a configural model using multigroup CFA (MGCFA) to check whether the factor structure varies across genders and countries. Chen [48] suggests a change of ≤−0.005 in CFI, supplemented by a change of ≥0.010 in RMSEA, as an indicator of non-invariance when the compared sample sizes are unequal. We also used composite reliability (CR > 0.60 is adequate) and average variance extracted (AVE > 0.50 is adequate, but AVE > 0.40 is still acceptable if CR > 0.60) statistics for confirmatory composite analysis (CCA) assessment [47,49].

Pearson’s correlation and multiple linear regression analysis (with life satisfaction as a dependent variable) were performed for associations between PPLIS, life satisfaction, stress, and depression to examine criterion validity. Additionally, an independent samples *t*-test was conducted to examine intergroup differences between emerging and non-emerging adults in PPLIS, life satisfaction, stress, and depression.

Descriptive statistics were performed using JASP ver. 0.16.1.0. for Windows. All structural analyses were conducted using IBM SPSS ver. 26 and AMOS ver. 26 software for Windows. Confirmatory composite analysis (CCA) was assessed using the AMOS ver. 26 plugin *Master Validity Tool* created by Gaskin et al. [50].

## 3. Results

### 3.1. Exploratory Factor Analysis

Initially, the parametric properties were examined (Table 1) in Sample 1 (*N* = 3230). All items showed good parametric properties, including that skewness and kurtosis ranged between ±1, and all correlations were significant on *p* < 0.001, with values above *r* = 0.30. 

The EFA with principal components extraction method was performed for Sample 1 of university students, and separately for Polish (*n* = 1581) and Ukrainian (*n* = 1649) participants, to replicate results in different countries. In the first step, the Kaiser–Mayer–Olkin (KMO) measure of adequacy and Bartlett’s test of sphericity were conducted. The results showed an average value of KMO = 0.76 for Polish and Ukrainian students. Furthermore, an adequate Bartlett’s test was found for the Polish sample χ^2^(6) = 1874.98, *p* < 0.001, and the Ukrainian group χ^2^(6) = 1864.21, *p* < 0.001, indicating that a correlation between the items in the PPLIS-4 and factor analysis is plausible. According to the Kaiser Criterion (eigenvalue greater than 1) and Cattell’s scree plot, the one-factor solution was found for Polish and Ukrainian university students. The factor loadings were 0.69, 0.73, 0.85, and 0.83 in the Polish sample; and 0.74, 0.76, 0.81, and 0.81 in the Ukrainian group for items PPLIS1, PPLIS2, PPLIS3, and PPLIS4, respectively. The first component explained 60.89% of perceived positive lockdown consequences in Poland and 60.83% in Ukraine. The next components explained, respectively, 16.46%, 14.40%, and 8.25% in the Polish sample, and 16.08%, 13.70%, and 9.40% in the Ukrainian group of university students.

### 3.2. Confirmatory Factor and Composite Analyses

**Figure 1:**
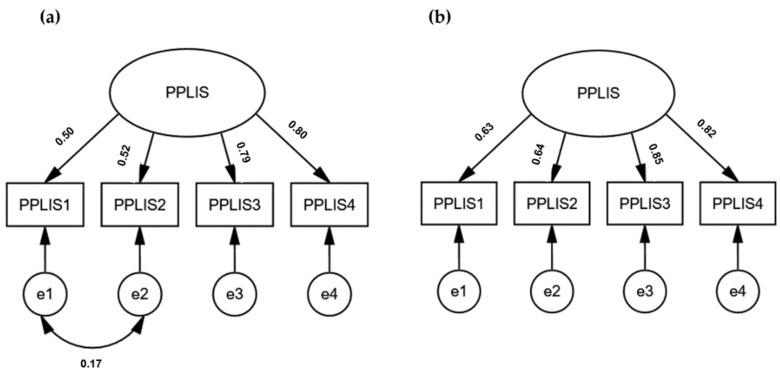
Results of Confirmatory Factor Analysis for (**a**) Sample 2 and (**b**) Sample 3. PPLIS = Perceived Positive Lockdown Impact Scale. Numbers by arrows represent standardized loading weights (β); e1–e4 = error variance.

The CFA was conducted for Sample 2 of Polish emerging adults (university students under 26, *N* = 448) and Sample 3 (Polish and Ukrainian adults aged above 25 years old, *N* = 652) separately. As shown in Figure 1a, the loading weight ranged between 0.50 and 0.80. The fit indices were excellent for Sample 2, χ^2^(1) = 2.13, *p* = 0.144, SRMR = 0.010, CFI = 0.997, RMSEA = 0.050 (90%CI = 0.000, 0.147). Considering the confirmatory composite analysis (CCA), Cronbach’s α = 0.76, CR = 0.754, and AVE = 0.445, suggesting that the structure is acceptable [49]. The CFA loadings for Sample 3 ranged between 0.63 and 0.85, as presented in Figure 1b. The fit indices were excellent for Sample 3, χ^2^(2) = 0.83, *p* = 0.661, SRMR = 0.005, CFI = 1.000, RMSEA = 0.000 (90%CI = 0.000, 0.060). The CCA was also adequate for Sample 3, including Cronbach’s α = 0.82, CR = 0.828, and AVE = 0.55. Configural multigroup measurement invariance was examined across genders and countries. The baseline model was compared to the unconstrained model for genders in Sample 2. The unconstrained model showed a good fit, χ^2^(2) = 1.966, *p* = 0.374, SRMR = 0.016, CFI = 0.100, RMSEA = 0.000 (90%CI = 0.000, 0.093), showing that the structure of PPLIS is invariant across genders. The configural MI was examined in Sample 3. The results repeated previous findings, demonstrating that the one-factor structure is invariant across gender, χ^2^(4) = 2.509, *p* = 0.643, SRMR = 0.006, CFI = 0.100, RMSEA = 0.000 (90%CI = 0.000, 0.048). Similarly, Polish and Ukrainian university students do not differ in factor structure, χ^2^(4) = 3.498, *p* = 0.478, SRMR = 0.015, CFI = 0.100, RMSEA = 0.000 (90%CI = 0.000, 0.056).

### 3.3. Criterion Validity

**Figure 2:**
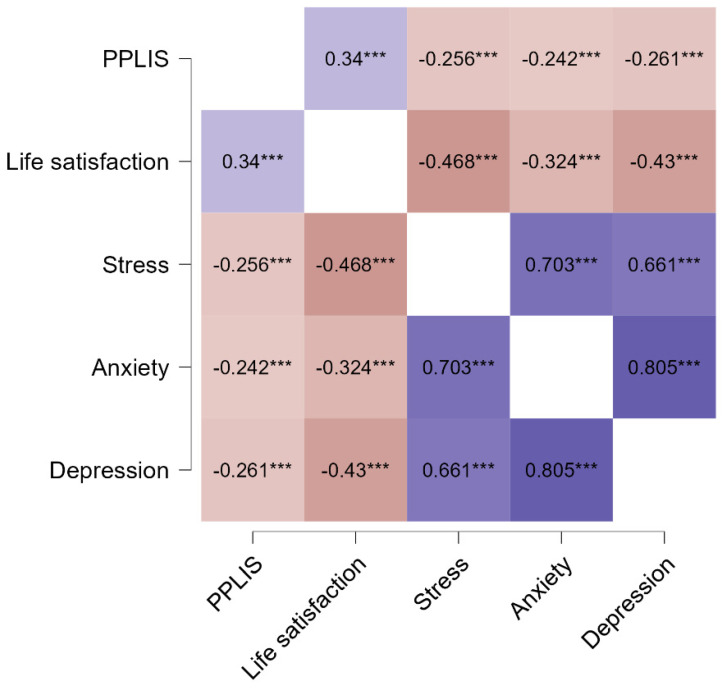
Pearson’s correlations between the perceived positive lockdown impact scale (PPLIS) and well-being dimensions: life satisfaction, perceived stress, anxiety, and depression. *N* = 3230, *** *p* < 0.001. Violet represents a positive correlation and red a negative correlation.

Criterion validity was examined using correlation and regression analysis to assess associations between the PPLIS and such variables as life satisfaction, perceived stress, self-rated physical health, anxiety, and depression. The analysis was performed for Sample 1 (Figure 2). Convergent validity was confirmed since the perceived positive impact of lockdown was related positively to life satisfaction. Discriminant validity was also supported by the significant negative association between the perceived positive impact of lockdown and mental health issues, like perceived stress, anxiety, and depression. 

Linear regression was conducted for life satisfaction as a dependent variable, and the PPLIS, perceived stress, anxiety, and depression, as predictors. All variables were significant predictors of life satisfaction, and the regression model explained 31% of life satisfaction variance, *R* = 0.56, *R*^2^ = 0.31, *F*(4, 3229) = 360.69, *p* < 0.001 (Table 2). 

The Student’s *t*-test was performed to explore differences between emerging and non-emerging adults in the sample of university students (*N* = 3230). The analysis showed that emerging adults scored significantly higher (with medium effect size) than non-emerging adults in perceived positive lockdown impact on life (Table 3). No significant differences were found between samples in life satisfaction, stress, anxiety, and depression.

## 4. Discussion

This study aimed to validate the scale of the perceived positive impact of lockdown. Several statistical tests were performed. The one-factor structure of the PPLIS-4 was confirmed twice in Polish and Ukrainian samples of university students using the EFA. Similarly, the construct was further validated using CFA twice in both emerging adults and non-emerging adult samples. The parametric properties of particular items and composite scores were good, and the one-factor model showed an excellent fit in both samples. Furthermore, the multigroup measurement invariance was supported across countries and genders, evidencing that the scale can be used in various cross-cultural contexts. Replicating our statistical analyses, we demonstrated that the four-item brief PPLIS-4 is valid and can be used in further studies. 

The assumption of associations between the perceived positive impact of lockdown on life and well-being dimensions was confirmed in this study. The positive association of PPLIS-4 was found with life satisfaction as a general cognitive dimension of well-being, relating to various areas of life. Additionally, a negative relationship was presented between PPLIS-4 and such mental health issues as perceived stress, anxiety, and depression. Moreover, a higher value was demonstrated for intercorrelation between PPLIS-4 and life satisfaction (*r* = 0.34) than between PPLIS-4 and mental health issues (*r* ranged from −0.24 to −0.26). Furthermore, the regression model showed that the perceived positive lockdown impact was a significant predictor of life satisfaction, even controlling for stress, anxiety, and depression. These results show that the perceived positive impact of lockdown is closer to well-being than mental health, confirming the convergent validation. However, longitudinal research is needed in the future to verify whether the perceived positive impact of lockdown is a predictor of well-being.

This study found significant differences between emerging and non-emerging adults in PPLIS-4, but not in life satisfaction, stress, anxiety, and depression. The results seem consistent with previous longitudinal findings, which showed that mental health problems did not increase during the pandemic among emerging adults from the Netherlands [22]. In the other study, Lithuanian and German emerging adults even presented a decrease in stress and anxiety symptoms, alongside an increase in positive mental health indices during the first wave of the COVID-19 pandemic compared to the pre-pandemic period [23]. More than 80% of emerging adults demonstrated positive changes indicating resilience. Although the differences were not examined between emerging and non-emerging adults in previous studies, the studies mentioned above seem to support the current results and Arnett’s [20] observations that emerging adults are more optimistic about the future than other populations, which may be a resilience factor preventing the deterioration of mental health and well-being during a crisis.

The strength of this study is that statistical analyses were conducted in a large sample size, replicating EFA and CFA results. Considering all three samples, the number of all participants in the study was 4370. However, the limitation of this study is that most of these individuals were emerging adults, so comparison with non-emerging adults should be treated with caution. The other limitation of this study is that the samples of emerging adults consisted exclusively of university students. More research is necessary for the future, with more balanced groups regarding emerging and non-emerging adults, and those studying and not. The present study compared only two countries: Poland and Ukraine. The PPLIS-4 should be validated in various countries to verify its pertinence. Additionally, we used life satisfaction and selected mental health symptoms to examine the criterion validity of the PPLIS-4. The other variables can be used in future studies to confirm convergent validation of the PPLIS-4, including various dimensions of well-being, optimism, hope, and resilience. Finally, the study was cross-sectional. Longitudinal research should be performed to examine the stability of the PPLIS-4 across various time points and to explore the predictive value of the perceived positive lockdown impact on life with individual differences in other positive and negative dimensions of mental health and well-being. This study is quantified and fails to explain the social and cultural reasons behind the perceived positive effects of the COVID-19 pandemic. Future research may use qualitative methods, such as interviews or case studies, to shed more light on this positive aspect of the COVID-19 pandemic.

## 5. Conclusions

The study showed that the PPLIS-4 is a valid instrument to measure the positive effects of the lockdown among emerging and non-emerging adults. The study showed good parametric properties of the scale and its invariance across countries and genders. Although more research is required to confirm the present results in cross-cultural studies, the tool can be used in the adult population during mandatory social isolation in the future. PPLIS-4 can be used to assess perceived positive life changes during the pandemic lockdown and can be modified in various therapeutic situations for people who are socially isolated due to chronic diseases or disabilities.

## Figures and Tables

**Table 1:** Descriptive statistics for perceived positive lockdown consequences scale (PPLIS) in Sample 1 (*N* = 3230).

Items	Range	*M*	*SD*	Skew.	Kurt.	Pearson’s Correlations			
						PPLIS1	PPLIS2	PPLIS3	PPLIS4
PPLIS1	1–7	4.42	1.89	−0.29	−0.94				
PPLIS2	1–7	3.23	1.93	0.50	−0.89	0.44 *** (0.41, 0.47)			
PPLIS3	1–7	3.88	1.93	0.06	−1.10	0.44 *** (0.41, 0.47)	0.48 *** (0.46, 0.51)		
PPLIS4	1–7	4.09	1.96	−0.06	−1.15	0.46 *** (0.43, 0.48)	0.49 *** (0.46, 0.52)	0.64 *** (0.62, 0.66)	
PPLIS Total score	4–28	15.62	6.07	0.04	−0.66	0.74 *** (0.72, 0.75)	0.77 *** (0.75, 0.78)	0.82 *** (0.80, 0.83)	0.83 *** (0.81, 0.84)

Note. PPLCS = perceived positive lockdown consequences scale. *** *p* < 0.001.

**Table 2:** Multiple linear regression for life satisfaction (*N* = 3230).

Variable	*B*	*SE B*	β	*t*	*p*
Intercept	25.30	0.40		62.83	<0.001
Stress	−0.30	0.02	−0.37	−17.57	<0.001
Anxiety	0.30	0.03	0.26	9.85	<0.001
Depression	−0.33	0.02	−0.34	−13.26	<0.001
PPLIS	0.22	0.02	0.22	14.43	<0.001

Note. PPLIS = Perceived Positive Lockdown Impact Scale.

**Table 3:** Student’s *t*-test for differences between emerging and non-emerging adults (*N* = 3230).

Variable	Emerging Adults (*n* = 3042)		Non-Emerging Adults (*n* = 188)		*t*(3228)	*p*	*d*
PPLIS	15.78	6.03	13.11	6.21	−5.87	<0.001	−0.44
Life satisfaction	21.74	6.05	21.52	6.19	−0.49	0.620	−0.04
Stress	20.49	7.41	20.42	7.53	−0.13	0.900	< 0.01
Anxiety	6.73	5.25	7.47	6.05	1.85	0.060	0.14
Depression	8.51	6.21	8.78	6.58	0.57	0.570	0.04

Note. PPLIS = Perceived Positive Lockdown Impact Scale.

## Data Availability

The datasets used and analyzed during the current study are available from the corresponding author upon reasonable request.

## Appendix A

**Table A1:** The Perceived Positive Lockdown Impact Scale (PPLIS-4).

I Think the Situation Related to the COVID-19 Pandemic Has Positively Affected My Life Through:		Completely						Completely
		Disagree						Agree
1	Spending more time with family	1	2	3	4	5	6	7
2	Spending more time with friends	1	2	3	4	5	6	7
3	Spending more time relaxing and on entertainment	1	2	3	4	5	6	7
4	Spending more time on personal development and interests (hobbies)	1	2	3	4	5	6	7

**Table A2:** Skala Postrzeganego Pozytywnego Wpływu Kwarantanny (SPPWK-4).

Uważam, że sytuacja związana z pandemią COVID-19, pozytywnie wpłynęła na moje życie poprzez:		Zdecydowanie						Zdecydowanie
		się nie zgadzam						się zgadzam
1	Spędzanie więcej czasu z rodziną	1	2	3	4	5	6	7
2	Spędzanie więcej czasu z przyjaciółmi	1	2	3	4	5	6	7
3	Poświęcanie więcej czasu na odpoczynek i rozrywkę	1	2	3	4	5	6	7
4	Poświęcanie więcej czasu na rozwój osobisty i zainteresowania (hobby)	1	2	3	4	5	6	7

**Table A3:** Шкала сприйняття позитивного впливу локдауну (ШСПВЛ-4).

Я вважаю, що ситуація, пов’язана з пандемією COVID-19, позитивно вплинула на моє життя, оскільки:		Повністю						Повністю
		не погоджуюся						погоджуюся
1	я проводжу більше часу з сім’єю	1	2	3	4	5	6	7
2	я проводжу більше часу з друзями	1	2	3	4	5	6	7
3	я витрачаю більше часу на відпочинок та розваги	1	2	3	4	5	6	7
4	я витрачаю більше часу на власний розвиток та зацікавлення (хобі)	1	2	3	4	5	6	7
